# Social Isolation Trajectories Spanning Childhood to Adulthood and Mortality Risk in CKM Syndrome: Evidence From CHARLS

**DOI:** 10.1002/brb3.71328

**Published:** 2026-03-30

**Authors:** Luyue Liang, Xinbo Wang, Shuaiqing Chen, Mingshen Lin

**Affiliations:** ^1^ Zhejiang Chinese Medical University Hangzhou China; ^2^ Department of Pediatrics, Lishui Municipal Central Hospital Fifth Affiliated Hospital of Wenzhou Medical University Lishui China; ^3^ Department of Clinical Laboratory Lishui Municipal Central Hospital Fifth Affiliated Hospital of Wenzhou Medical University Lishui China; ^4^ Department of Cardiology Lishui Hospital of Traditional Chinese Medicine Lishui China

**Keywords:** all‐cause mortality, cardiovascular‐kidney‐metabolic syndrome, China health and retirement longitudinal study, cystatin C, social isolation

## Abstract

**Background:**

Social isolation is increasingly recognized as a critical determinant of health. This study aimed to systematically evaluate the association between social isolation trajectories spanning childhood to adulthood and all‐cause mortality in patients with cardiovascular‐kidney‐metabolic (CKM) syndrome stages 1–4 and to investigate the potential mediating role of renal function.

**Methods:**

We analyzed data from 5019 participants aged 45 years or older with CKM stages 1–4 from the China Health and Retirement Longitudinal Study (CHARLS). Four life‐course social isolation trajectories were identified: no isolation, childhood‐only, adulthood‐only, and persistent isolation. Cox proportional hazards models and exploratory mediation analyses were used to assess all‐cause mortality risk and potential mediating effects. Additionally, Kaplan‐Meier curves, subgroup analyses, and sensitivity analyses were performed.

**Results:**

Persistent isolation was linked to an elevated likelihood of all‐cause mortality (HR = 1.92, 95% CI: 1.25–2.97). The mediation analysis suggested that cystatin C contributed to the pathway linking persistent isolation with mortality, explaining 6.31% of the overall effect (*p* < 0.05).

**Conclusions:**

Persistent social isolation shows a clear association with elevated mortality risk among middle‐aged and older Chinese individuals living with CKM syndrome, and this association is partially explained by cystatin C levels, serving as a potential mechanistic link. Integrating long‐term psychosocial assessment into CKM risk stratification and management is crucial.

## Graphical abstract

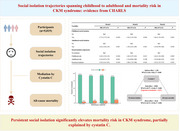

## 1 Introduction

Against a backdrop of escalating obesity, widespread diabetes, and rapid population aging, the cardiovascular‐kidney‐metabolic (CKM) syndrome has emerged as a critical global health threat (Aggarwal et al. 2024). In 2023, the American Heart Association (AHA) formally articulated the concept of CKM syndrome, underscoring the intricate pathophysiological interplay linking metabolic derangements, chronic kidney disease (CKD), and cardiovascular dysfunction (Ndumele et al. 2023). These multisystem interactions accelerate atherosclerotic progression and substantially elevate mortality risk (Ostrominski et al. 2023). The situation is particularly acute in China, where recent epidemiological data indicate that nearly 90 percent of middle‐aged and older adults meet the diagnostic criteria for CKM syndrome (Hui Zhang et al. 2025). Such an extraordinary prevalence reflects the growing accumulation of metabolic risk within the population and signals an impending surge in cardiovascular and renal disease burden, especially in advanced stages where the risks of all‐cause and cardiovascular mortality rise sharply (Congyi Zheng et al. 2025). Despite this, current clinical strategies still focus predominantly on lifestyle modification and pharmacologic management. This biomedical approach increasingly reveals its limitations, as it overlooks the broader psychosocial and environmental contexts in which individuals live. These social determinants of health exert profound and lasting influences on health trajectories across the life span and are essential considerations for effective CKM management.

Social isolation, defined as an objective lack of social connection or support, has been reframed by the World Health Organization (WHO) as an urgent medical concern rather than a purely social issue (Holt‐Lunstad and Perissinotto 2023). In a 2023 advisory, the United States Surgeon General cautioned that the mortality risk associated with social disconnection rivals that attributed to smoking and obesity (Office of the Surgeon General (OSG) 2023). Evidence indicates that persistent isolation functions as a chronic stressor that triggers systemic inflammatory activation and neuroendocrine dysregulation, thereby accelerating pathological injury across multiple organ systems (Cené et al. 2022; Liang et al. 2023). Although numerous studies have linked social isolation occurring in either childhood or adulthood to elevated mortality, these investigations have largely relied on single time‐point assessments (Wang et al. 2023; Matthews and Li 2023). Such an approach fails to capture the inherently dynamic nature of isolation as it evolves over the life course. According to life‐course epidemiologic theory, social withdrawal or experiences of bullying during childhood may become biologically embedded and interact with adverse exposures in adulthood, resulting in a cumulative disease burden that exceeds the simple sum of isolated risks (Kim et al. 2023; Suglia and April‐Sanders 2024; Barrett‐Young et al. 2023). However, evidence remains scarce regarding how social isolation trajectories spanning childhood to adulthood shape long‐term survival, particularly among individuals with CKM syndrome who represent a highly vulnerable population.

In addition, the kidney, a central organ within the CKM axis, is particularly vulnerable to the wear imposed by chronic psychosocial stress (Seery and Buchanan 2022; Park and Kang 2025). Prior evidence suggests that prolonged stress responses may hasten renal functional decline through disturbances in neurohumoral regulation and low‐grade inflammatory processes, changes that often precede the clinical manifestation of kidney disease (Koga et al., 2022; Li et al., 2024). Yet it is still unclear whether the detrimental impact of social isolation contributes to mortality through specific patterns of renal impairment.

Against this background, the study drew on nine years of nationally representative longitudinal data from the China Health and Retirement Longitudinal Study (CHARLS). We posited that persistent social isolation would be strongly associated with elevated mortality risk and that specific renal biomarkers might capture the cross‐sectional mechanistic pathways linking this relationship. By delineating the cumulative impact of life‐course social isolation on individuals with CKM stages 1 to 4, the findings aim to provide robust epidemiologic evidence and conceptual grounding for more precise risk stratification and early intervention strategies in this high‐risk population.

## 2 Methods

### 2.1 Study Design and Participants

This analysis drew on data from CHARLS, a nationally representative prospective cohort established to monitor demographic, socioeconomic, and health trajectories among Chinese adults aged 45 years or older (Zhao et al. 2014). Following its initiation in 2011–2012, subsequent follow‐up assessments were conducted in 2013, 2015, 2018, and 2020. A detailed life‐course survey was incorporated in 2014, and venous blood samples were collected in 2012 and 2015 for biomarker measurements. All interviews were administered by trained personnel using a computer‐assisted personal interviewing system. The study protocol received approval from the Peking University Biomedical Ethics Review Committee (approval number IRB00001052‐11015), and written informed consent was obtained from all participants at enrollment.

**FIGURE 1:**
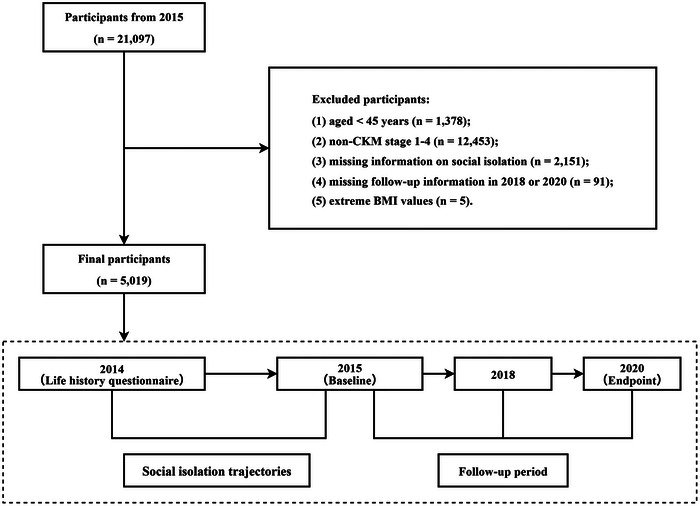
Flowchart of participant selection and schematic of the study design.

To align the adult assessments with childhood history collected in 2014, the 2015 survey wave was designated as the analytical baseline, and outcomes were tracked through the 2018 and 2020 waves. From all individuals who participated in the 2015 assessment, a total of 5019 participants were ultimately included in the analysis (Figure 1). Participants were excluded if they fulfilled any of the following conditions: age younger than 45 years; absence of CKM stages 1 to 4; incomplete information on childhood or adulthood social isolation; unavailable survival status or death date; or biologically implausible body mass index (BMI) values of 80 kg/m^2^ or higher. Baseline characteristics of included versus excluded participants are presented in Supplementary Table S1.

### 2.2 Classification Criteria for CKM Syndrome

Participants were classified into CKM syndrome stages according to the most recent criteria issued by the AHA. The staging framework spans from stage 0, which reflects optimal cardiometabolic status, to stage 4, which represents established clinical cardiovascular disease. Intermediate stages were defined as follows. Stage 1 denotes adipose tissue dysfunction manifested by obesity or impaired glucose regulation. Stage 2 encompasses major metabolic risk factors, including hypertension, type 2 diabetes, hypertriglyceridemia, metabolic syndrome, or moderate CKD. Stage 3 represents subclinical cardiovascular or renal pathology, characterized by a 10‐year cardiovascular risk of 20 percent or higher estimated using the Framingham Risk Score or the presence of severe CKD. The complete diagnostic algorithm is provided in Supplementary Table S2.

### 2.3 Exposure Assessment

We characterized social isolation across the life course by creating a composite trajectory variable that integrated information from both childhood and adulthood. Childhood isolation was derived from the 2014 life‐course survey, which operationalized Caspi's framework of social withdrawal and exclusion (Caspi et al. 2006). Four items were assessed: having a group of friends and feeling lonely, which captured withdrawal, and being bullied by neighbors or school peers, which reflected exclusion (Wang et al. 2023). Each item was scored on a 3‐point scale. The item on having friends was reverse coded (often = 0; sometimes = 1; rarely or never = 2), whereas the remaining items were scored in the forward direction (rarely or never = 0; sometimes = 1; often = 2). Scores were summed to yield a cumulative index ranging from 0 to 8, and values of 2 or higher were classified as childhood social isolation. Adulthood isolation was assessed at the 2015 baseline using a composite index comprising four binary indicators: being unmarried, living alone, absence of weekly contact with children, and lack of social participation (Song et al., 2023). A cumulative score of 2 or higher (range: 0–4) indicated adulthood social isolation. Consistent with prior research, we derived four mutually exclusive trajectories of social isolation by cross‐classifying isolation status across the two life stages (Lay‐Yee et al., 2021). These trajectories were defined as no isolation, childhood‐only isolation, adulthood‐only isolation, and persistent isolation.

### 2.4 Mediating Variables

To investigate potential biological mechanisms linking social isolation trajectories to all‐cause mortality, we included two key biomarkers indicative of renal impairment, serum creatinine (SCr) and cystatin C, as candidate mediators. Analytically, SCr concentrations were quantified using the interference‐corrected Jaffe method, whereas cystatin C levels were measured by particle‐enhanced immunonephelometry.

### 2.5 Outcome Definition

All‐cause mortality was ascertained using data from the 2018 and 2020 follow‐up assessments. For deceased participants, trained field interviewers verified death status and date through proxy interviews with family members or other knowledgeable informants (Chen et al. 2023; Ou et al. 2025). Survival time was calculated as the interval from the 2015 baseline interview to the date of death or the date of the last follow‐up, whichever occurred first.

### 2.6 Covariates

We adjusted analyses for a series of baseline covariates selected based on literature review and clinical relevance to account for potential confounding. Demographic characteristics included age, sex, educational attainment (categorized as below elementary, elementary, and above elementary), and residential area (urban or rural). Lifestyle factors comprised smoking and alcohol consumption. Clinical health status was characterized using BMI, CKM stage, and the presence of hypertension, diabetes, and dyslipidemia. Depressive symptoms were assessed with the 10‐item Center for Epidemiologic Studies Depression Scale (CES‐D 10), with a total score of 10 or higher indicating clinically significant depression. Early‐life environment was evaluated using childhood family economic status and self‐rated health from the life‐course survey, which were recoded into three ordered categories: poor, fair, and good. The proportion of missing data for these covariates is presented in Supplementary Figure S1.

### 2.7 Statistical Analysis

Baseline characteristics were reported stratified by the four social isolation trajectories. Continuous variables are presented as mean ± standard deviation and compared using analysis of variance (ANOVA), whereas categorical variables are expressed as counts and percentages, with differences assessed using Pearson's chi‐square test or Fisher's exact test as appropriate. To minimize bias and maximize data utilization, missing covariate data was imputed using multiple imputation by chained equations (MICE), generating five complete datasets, with results pooled according to Rubin's rules.

Survival differences across social isolation trajectories were initially visualized using Kaplan–Meier curves and evaluated with log‐rank tests. Multivariable Cox proportional hazards models were then constructed to estimate hazard ratios (HRs) and 95% confidence intervals (CIs) for childhood isolation, adulthood isolation, and social isolation trajectories, using the no‐isolation group as the reference. A hierarchical modeling approach was adopted: Model 1 was unadjusted; Model 2 adjusted for age, sex, education, and residence; and Model 3 further adjusted for early‐life environment, lifestyle factors, depression, and CKM stage.

To rigorously evaluate the robustness of our primary findings, a series of subgroup and sensitivity analyses were conducted. Subgroup analyses stratified the fully adjusted population by sex, residence, and depression, with interaction tests employed to assess potential effect modification. Two key sensitivity analyses were performed: first, to reduce the possibility of reverse causation, we reconstructed the Cox regression models after removing individuals who died during the first three years of follow‐up as part of a lagged sensitivity assessment; second, to assess the reliability of the imputation procedure, a complete‐case analysis excluding observations with missing values was conducted.

Finally, we performed an exploratory mediation analysis to investigate potential biological mechanisms linking persistent social isolation to all‐cause mortality. Adjusting for all covariates in model 3, indirect effects and their proportion of the total effect were estimated using a Bayesian approach based on 1,000 simulations.

All statistical analyses were conducted using R software (version 4.2.2). Statistical significance was defined as a two‐sided *p‐*value less than 0.05.

## 3 Results

### 3.1 Basic Characteristics of Participants

The final analytical cohort comprised 5019 participants with a mean age of 60.1 ± 8.5 years, of whom 54.7% were male. The distribution across social isolation trajectories was as follows: no isolation (*n* = 3230, 64.4%), childhood‐only isolation (*n* = 1215, 24.2%), adulthood‐only isolation (*n* = 342, 6.8%), and persistent isolation (*n* = 232, 4.6%). As detailed in Table 1, compared with the no‐isolation group, participants in the persistent isolation group were older, had lower educational attainment, and were more likely to reside in rural areas. Regarding early‐life environment, the persistent isolation group had the highest proportions of poor childhood economic status (47.0%) and poor self‐rated health during childhood (12.9%). Notably, although the burden of chronic metabolic diseases was broadly comparable across groups, substantial differences were observed in mental health outcomes. Specifically, the prevalence of depression reached 46.1% in the persistent isolation group, markedly higher than the 24.3% observed in the no‐isolation group.

**TABLE 1:** Baseline characteristics of participants stratified by social isolation trajectories.

Variables	Total	No isolation	Childhood only (*n* = 1215)	Adulthood only (*n* = 342)	Persistent isolation (*n* = 232)	*P*
	(*n* = 5019)	(*n* = 3230)				
**Age, years**	60.1 ± 8.5	59.3 ± 8.3	60.8 ± 8.6	62.5 ± 9.2	64.2 ± 9.2	< 0.001
**BMI, kg/m^2^**	24.3 ± 4.0	24.5 ± 3.9	24.1 ± 4.3	23.8 ± 3.5	23.4 ± 3.4	< 0.001
**Sex, *n* (%)**						0.056
Female	2274 (45.3%)	1,483 (45.9%)	513 (42.2%)	168 (49.1%)	110 (47.4%)	
Male	2745 (54.7%)	1747 (54.1%)	702 (57.8%)	174 (50.9%)	122 (52.6%)	
**Education, *n* (%)**						< 0.001
Below elementary	1329 (26.5%)	701 (21.7%)	435 (35.8%)	91 (26.6%)	102 (44.0%)	
Elementary	1461 (29.1%)	915 (28.3%)	363 (29.9%)	104 (30.4%)	79 (34.1%)	
Above elementary	2229 (44.4%)	1614 (50.0%)	417 (34.3%)	147 (43.0%)	51 (22.0%)	
**Residence, *n* (%)**						< 0.001
Urban	1083 (21.6%)	779 (24.1%)	209 (17.2%)	64 (18.7%)	31 (13.4%)	
Rural	3936 (78.4%)	2451 (75.9%)	1006 (82.8%)	278 (81.3%)	201 (86.6%)	
**Smoking, *n* (%)**						0.622
No	3453 (68.8%)	2239 (69.3%)	830 (68.3%)	226 (66.1%)	158 (68.1%)	
Yes	1566 (31.2%)	991 (30.7%)	385 (31.7%)	116 (33.9%)	74 (31.9%)	
**Drinking, *n* (%)**						<0.001
No	3055 (60.9%)	1895 (58.7%)	774 (63.7%)	232 (67.8%)	154 (66.4%)	
Yes	1964 (39.1%)	1335 (41.3%)	441 (36.3%)	110 (32.2%)	78 (33.6%)	
**CKM, *n* (%)**						0.791
Stage 1	382 (7.6%)	257 (8.0%)	85 (7.0%)	22 (6.4%)	18 (7.8%)	
Stage 2	966 (19.2%)	619 (19.2%)	235 (19.3%)	67 (19.6%)	45 (19.4%)	
Stage 3	2551 (50.8%)	1,613 (49.9%)	643 (52.9%)	175 (51.2%)	120 (51.7%)	
Stage 4	1120 (22.3%)	741 (22.9%)	252 (20.7%)	78 (22.8%)	49 (21.1%)	
**Hypertension, *n* (%)**						0.929
No	3302 (65.8%)	2134 (66.1%)	792 (65.2%)	226 (66.1%)	150 (64.7%)	
Yes	1717 (34.2%)	1096 (33.9%)	423 (34.8%)	116 (33.9%)	82 (35.3%)	
**Diabetes, *n* (%)**						0.970
No	4486 (89.4%)	2885 (89.3%)	1090 (89.7%)	304 (88.9%)	207 (89.2%)	
Yes	533 (10.6%)	345 (10.7%)	125 (10.3%)	38 (11.1%)	25 (10.8%)	
**Dyslipidemia, *n* (%)**						0.172
No	3907 (77.8%)	2515 (77.9%)	928 (76.4%)	273 (79.8%)	191 (82.3%)	
Yes	1,112 (22.2%)	715 (22.1%)	287 (23.6%)	69 (20.2%)	41 (17.7%)	
**Depression, *n* (%)**						< 0.001
No	3566 (71.1%)	2444 (75.7%)	767 (63.1%)	230 (67.3%)	125 (53.9%)	
Yes	1453 (28.9%)	786 (24.3%)	448 (36.9%)	112 (32.7%)	107 (46.1%)	
**Childhood economic status, *n* (%)**						< 0.001
Good	487 (9.7%)	343 (10.6%)	85 (7.0%)	43 (12.6%)	16 (6.9%)	
Fair	2662 (53.0%)	1835 (56.8%)	541 (44.5%)	179 (52.3%)	107 (46.1%)	
Poor	1870 (37.3%)	1052 (32.6%)	589 (48.5%)	120 (35.1%)	109 (47.0%)	
**Childhood health status, n (%)**						< 0.001
Good	1934 (38.5%)	1317 (40.8%)	401 (33.0%)	128 (37.4%)	88 (37.9%)	
Fair	2500 (49.8%)	1581 (48.9%)	622 (51.2%)	183 (53.5%)	114 (49.1%)	
Poor	585 (11.7%)	332 (10.3%)	192 (15.8%)	31 (9.1%)	30 (12.9%)	

Data are presented as mean ± standard deviation (SD) or number (%), as appropriate.

The *p*‐value is based on analysis of variance (ANOVA), chi‐square test, or Fisher's exact test.

Abbreviations: BMI, body mass index; CKM, cardiovascular‐kidney‐metabolic syndrome.

### 3.2 Associations of Social Isolation With All‐cause Mortality

During follow‐up, a total of 284 deaths (5.6%) were documented. As shown in Supplementary Figure S2, mortality varied markedly across social isolation trajectories: the no‐isolation group had the lowest incidence (4.5%), followed by childhood‐only isolation (7.5%) and adulthood‐only isolation (6.7%), with the highest burden observed in the persistent isolation group (10.8%). Kaplan‐Meier analysis confirmed this pattern, demonstrating significantly lower cumulative survival in the persistent isolation cohort compared with all other trajectory groups (log‐rank test *p* < 0.001; Supplementary Figure S3).

Table 2 presents detailed results from Cox proportional hazards regression analyses. Diagnostic checks prior to modeling indicated no evidence of multicollinearity, with variance inflation factors (VIFs) for all covariates below 2 (Supplementary Table S3). Additionally, Schoenfeld residuals confirmed that both the global test (*p* = 0.526) and individual covariates satisfied the proportional hazards assumption (Supplementary Table S4). When examining social isolation at distinct life stages, childhood isolation was consistently associated with higher all‐cause mortality across all models: model 1 (HR = 1.73, 95% CI: 1.37–2.19), model 2 (HR = 1.42, 95% CI: 1.12–1.81), and model 3 (HR = 1.46, 95% CI: 1.14–1.87). In contrast, adulthood isolation was significantly associated with mortality in model 1 (HR = 1.61, 95% CI: 1.18–2.19) and model 2 (HR = 1.38, 95% CI: 1.01–1.89), but the association attenuated and lost statistical significance in the fully adjusted model 3 (*p* = 0.056). Regarding social isolation trajectories, participants with persistent isolation exhibited a 150% higher mortality in model 1 compared with the no‐isolation group (HR = 2.50, 95% CI: 1.64–3.83). Although the magnitude of this association was slightly attenuated in model 2 (HR = 1.88, 95% CI: 1.22–2.88), it remained statistically significant in the fully adjusted model 3, corresponding to a 92% increased risk of death (HR = 1.92, 95% CI: 1.25–2.97). Notably, the childhood‐only isolation trajectory also retained a significant association with elevated mortality in the fully adjusted model (HR = 1.44, 95% CI: 1.10–1.89), whereas adulthood‐only isolation was not statistically significant (*p* = 0.243).

**TABLE 2:** Association of social isolation at different life stages and trajectories with all‐cause mortality.

Variables	Model 1		Model 2		Model 3	
	HR (95% CI)	*P*	HR (95% CI)	*P*	HR (95% CI)	*P*
**Childhood social isolation**						
No	Ref		Ref		Ref	
Yes	1.73 (1.37–2.19)	< 0.001	1.42 (1.12–1.81)	0.004	1.46 (1.14–1.87)	0.003
**Adulthood social isolation**						
No	Ref		Ref		Ref	
Yes	1.61 (1.18–2.19)	0.003	1.38 (1.01–1.89)	0.043	1.36 (0.99–1.86)	0.056
**Social isolation trajectories**						
No isolation	Ref		Ref		Ref	
Childhood only	1.69 (1.30–2.19)	< 0.001	1.40 (1.07–1.83)	0.013	1.44 (1.10–1.89)	0.009
Adulthood only	1.51 (0.97–2.35)	0.065	1.34 (0.86–2.08)	0.198	1.30 (0.84–2.03)	0.243
Persistent isolation	2.50 (1.64–3.83)	< 0.001	1.88 (1.22–2.88)	0.004	1.92 (1.25–2.97)	0.003

Model 1: Unadjusted.

Model 2: Adjust for Age, Sex, Education, Residence.

Model 3: Adjust for Age, Sex, Education, Residence, Smoking, Drinking, CKM, Depression, Childhood economic status, Childhood health status.

### 3.3 Subgroup and Sensitivity Analyses

**FIGURE 2:**
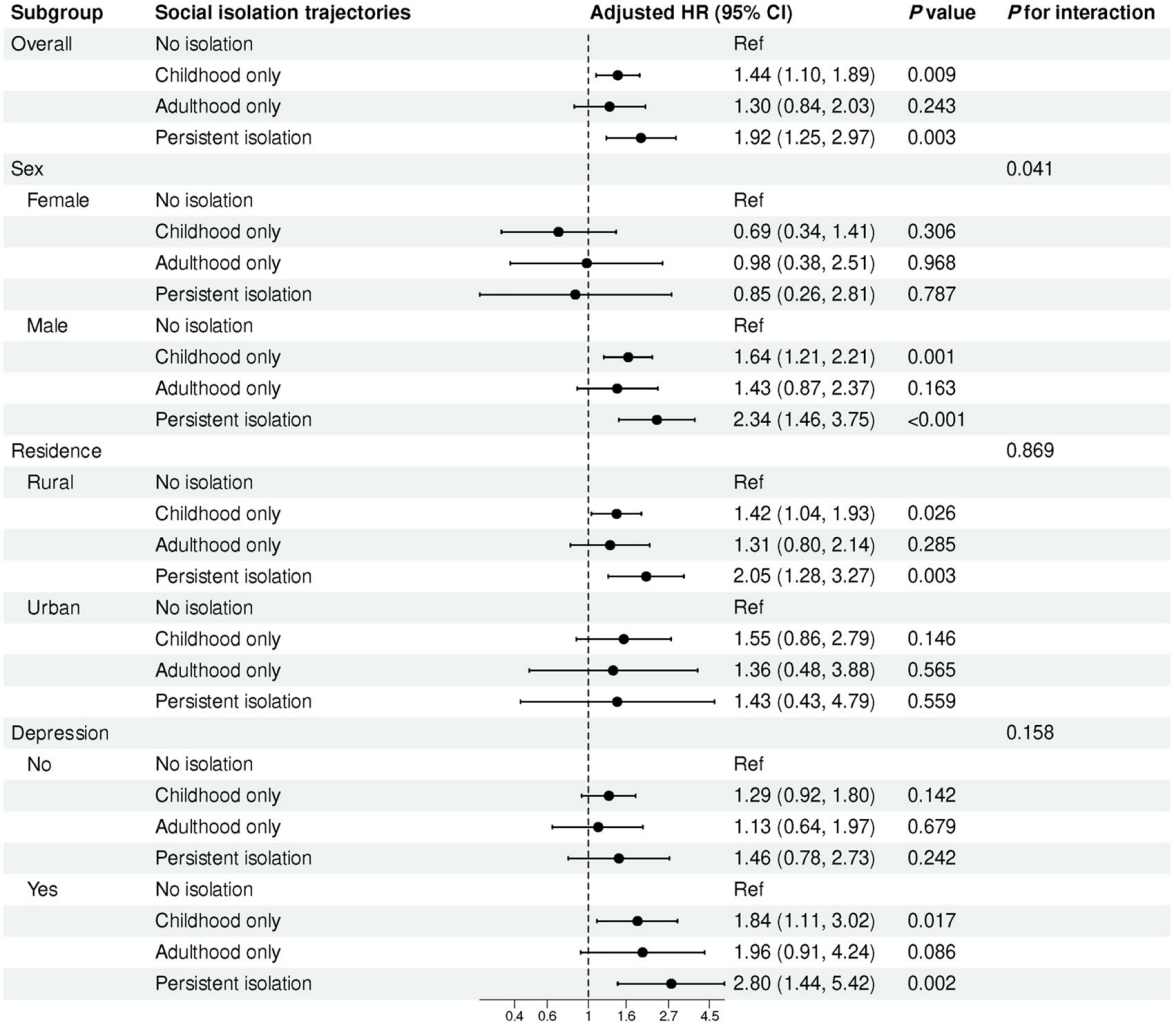
Subgroup analyses of the association between social isolation trajectories and all‐cause mortality.

Subgroup analyses showed that across groups defined by residence and depression, the relationship between social isolation trajectories and all‐cause mortality remained broadly consistent (Figure 2). However, a significant sex‐specific difference was observed (*P* for interaction = 0.041). Specifically, the detrimental effect of persistent isolation was primarily driven by male participants, whose mortality risk was markedly elevated (HR = 2.34, 95% CI: 1.46–3.75), whereas no significant association was detected among female participants (*p* = 0.787).

In the 3‐year lagged analysis, the association between persistent isolation and all‐cause mortality remained statistically significant (HR = 1.72, 95% CI, 1.19–2.64) (Supplementary Table S5). Additionally, complete‐case analysis, conducted after excluding participants with missing data, produced results highly consistent with the main analysis based on multiple imputation, with a hazard ratio of 1.77 (95% CI: 1.12–2.80) for the persistent isolation group (Supplementary Table S6).

### 3.4 Mediation Analysis

**FIGURE 3:**
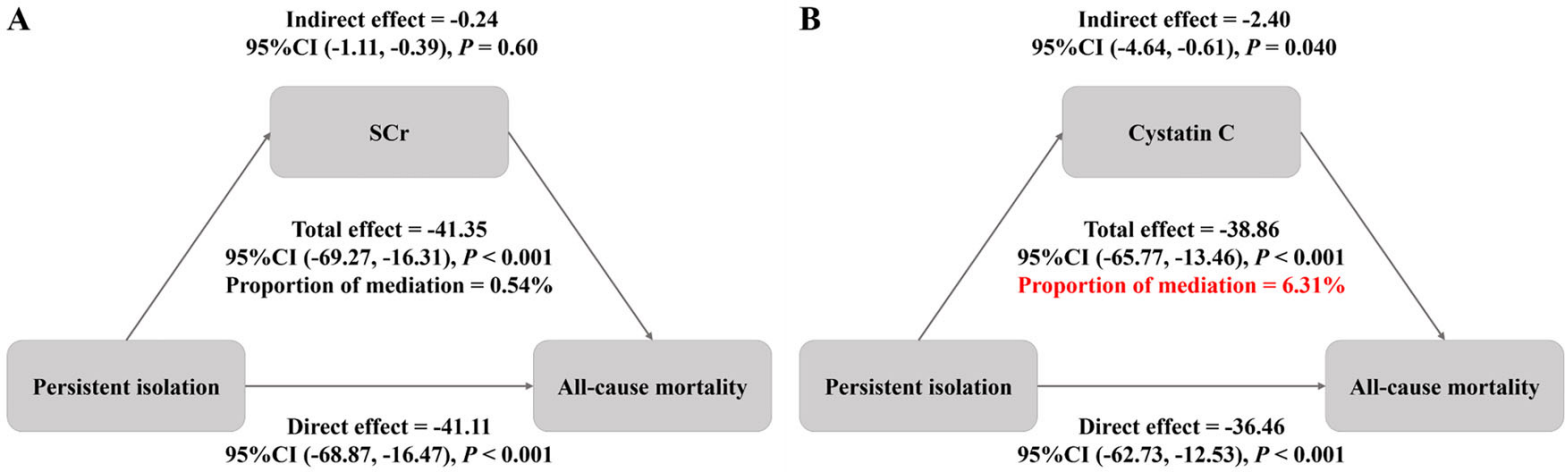
Mediation analysis of the association between persistent social isolation and all‐cause mortality. The diagrams illustrate the mediating roles of serum creatinine (A) and cystatin C (B).

Exploratory mediation analysis indicated that cystatin C partially explained the association between persistent social isolation and all‐cause mortality, accounting for 6.31% of the total observed effect (*p* < 0.05). In contrast, SCr did not demonstrate a statistically significant indirect effect, explaining only 0.54% of the association (*p* = 0.60) (Figure 3).

## 4 Discussion

This study provides the first comprehensive evaluation of life‐course social isolation trajectories and their association with all‐cause mortality among middle‐aged and older adults with CKM stages 1–4 in China, while also exploring the potential mediating role of renal dysfunction. Four distinct social isolation trajectories were identified, with individuals experiencing persistent isolation facing the highest mortality risk. Cystatin C was found to partially mediate this association. Collectively, these findings deepen our understanding of the long‐term harm resulting from social isolation in the context of CKM, offering empirical evidence to enhance early risk stratification and to inform targeted interventions along key pathophysiological pathways.

Recent evidence indicates that the dynamic evolution of social isolation may have greater relevance to health outcomes than baseline status alone. For example, Zhang and colleagues examined changes in social isolation in two prospective cohorts and their association with frailty in older adults: in the CHARLS cohort, persistent isolation was significantly associated with increased frailty risk (HR 1.2, 95% CI 1.1–1.4), whereas in the SHARE cohort, the risk was even more pronounced (HR = 1.4, 95% CI: 1.2–1.6) (2025). Lay‐Yee et al. constructed life‐course social isolation trajectories from childhood to midlife, demonstrating that persistent isolation was strongly linked to depression in adulthood (OR = 4.20, 95% CI: 1.84–9.61) (Lay‐Yee et al. 2023). Notably, Guo et al., using CHARLS data, found that middle‐aged and older adults experiencing persistent social isolation had a 45% higher risk of cardiovascular disease (HR = 1.45, 95% CI, 1.13–1.85) (2023). Our study extends this dynamic perspective to the vulnerable population of individuals with CKM stages 1–4. Compared with the general population, CKM patients are already at a critical threshold of multisystem metabolic dysregulation and reduced physiological reserve. In this context, persistent social isolation may no longer be merely a psychological or lifestyle burden; rather, it can act as a secondary hit, overwhelming residual compensatory mechanisms through cumulative neuroendocrine dysregulation and systemic inflammatory load, thereby contributing to increased mortality.

In addition, it is noteworthy that our study observed a significant association between childhood‐only isolation and increased mortality risk. This finding aligns with the concept of biological embedding, whereby adversity during critical developmental periods may induce lasting physiological imprints through remodeling of the neuroendocrine axis and epigenetic modifications (Yang et al. 2021; Copeland et al. 2022). Consequently, individuals may retain heightened vulnerability even if their social environment improves in adulthood. However, this result should be interpreted with caution, as the association did not reach statistical significance in the complete‐case sensitivity analysis, suggesting that the effect of childhood isolation, while enduring, may be weaker than that of persistent life‐course isolation or more susceptible to selection bias.

Our study also identified significant sex differences in the association between social isolation trajectories and mortality. The male‐specific vulnerability may reflect sociocultural and behavioral mechanisms. Sociological evidence indicates that men often rely predominantly on spouses for social support, rendering them more susceptible when isolated, whereas women typically maintain broader networks of relatives and friends that provide more resilient emotional support (Umberson et al. 2022; Cholankeril et al. 2023; Uhing et al. 2021). Additionally, men are more likely to adopt maladaptive coping strategies in response to psychosocial stress (Prowse et al. 2021; Alpers et al. 2024). Finally, sex differences in hormonal regulation of stress‐response systems may exacerbate physiological damage in men under chronic social stress (Hodes et al. 2024; Babcock et al. 2022).

Previous studies have demonstrated that social isolation, as a chronic stressor, can activate the hypothalamic‐pituitary‐adrenal (HPA) axis, resulting in sustained elevations of cortisol and subsequently inducing systemic inflammation and metabolic dysregulation (Zilioli and Jiang 2021; Sharma et al. 2021). Importantly, our mediation analysis further suggests that the cumulative burden of social stress may increase mortality risk in part through detrimental effects on renal function. In this study, cystatin C accounted for 5.92% of the observed association, whereas conventional SCr did not show a significant mediating effect. This discrepancy likely reflects differences in biomarker characteristics: SCr is influenced by muscle mass, which is often reduced in socially isolated older adults, potentially masking kidney impairment; in contrast, cystatin C is independent of muscle mass and closely associated with chronic low‐grade inflammation. Social isolation–induced microinflammation may lead to endothelial injury in the microvasculature and early subclinical declines in glomerular filtration rate (Kanbay et al. 2023). Thus, cystatin C not only reflects glomerular filtration function but is also closely linked to systemic inflammatory responses and early microvascular injury (Sluiter et al., 2024). Given that psychosocial stress can impact health via inflammatory and vascular pathways, cystatin C may partially capture these biological processes, serving as a key mechanistic link between social isolation and mortality risk (West et al. 2022). Crucially, while chronic kidney disease (CKD) severity is already embedded as a structural component within the CKM staging framework, elevated cystatin C captures an incremental pathophysiological dimension. It serves as a sensitive molecular sensor for the chronic low‐grade systemic inflammation and early subclinical microvascular endothelial injury driven by chronic psychosocial stress.

The findings of this study carry important implications for clinical risk stratification and public health practice. Assessment of social isolation can be accomplished through simple, low‐cost questionnaires, suggesting its potential utility as a practical tool to identify high‐risk individuals in primary care settings. Given the association of persistent isolation trajectories with the highest mortality risk, our results imply that longitudinal monitoring of psychosocial dynamics may provide prognostic information beyond that obtained from single‐timepoint assessments. For patients with CKM, clinicians should consider not only biomedical indicators but also the individual's life‐course social support history. Nevertheless, large‐scale prospective studies are needed to validate these findings and to determine whether interventions targeting social isolation and its downstream pathways can effectively reduce long‐term mortality risk.

We acknowledge several limitations. First, as an observational study, causality between social isolation and all‐cause mortality cannot be definitively established. Although multivariable adjustment and sensitivity analyses were employed to rigorously control for confounding, residual bias from unmeasured factors, such as genetic predisposition, cannot be completely excluded. Second, childhood social isolation was assessed retrospectively in adulthood, inherently introducing the possibility of recall bias. Although the CHARLS life‐course survey utilized life history calendar techniques to improve recall accuracy, and our fully adjusted models meticulously controlled for baseline depression and CKM severity to partially offset this mood‐congruent reporting bias, residual recall inaccuracies may still influence the observed associations. Furthermore, our assessment of adulthood social isolation relied on structural indicators. In the Chinese cultural context, these structured survey variables may still imperfectly capture the richness and complexity of informal social support, potentially leading to some degree of misclassification. Third, the sample was restricted to middle‐aged and older adults in China, which limits the generalizability of the findings to populations with different sociocultural backgrounds or age groups. Fourth, the exploratory mediation analysis is limited by temporal ordering constraints. Specifically, adulthood social isolation and cystatin C were measured concurrently during the 2015 baseline wave. Although the mortality outcome was prospectively tracked, the lack of a strict longitudinal sequence between the exposure and the mediator prevents the establishment of a definitive causal chain.

## 5 Conclusion

In conclusion, the study found that life‐course trajectories of social isolation are significantly linked to elevated all‐cause mortality among middle‐aged and older adults with CKM in China, with cystatin C acting as a potential mechanistic link. These results highlight the potential value of sustained monitoring of psychosocial changes.

## Author Contributions

L. L. and M. L. participated in data collection and drafting and editing of the paper. X. W. and S. C. participated in data analysis and revision of the paper. All authors saw and approved the final version, and no other person made a substantial contribution to the paper.

## Funding

This work was supported by the Zhejiang Province Traditional Chinese Medicine Science and Technology Plan Project (project number 2025ZL616). The funding body did not participate in the design of the study, data collection, analysis, interpretation of data, or writing the manuscript.

## Ethics Statement

This study was conducted in accordance with the Declaration of Helsinki. CHARLS was approved by the Institutional Review Board of Peking University (approval number: IRB00001052‐11015 for the household survey and IRB00001052‐11014 for blood samples).

## Consent

Written informed consent was obtained from all the participants.

## Conflicts of Interest

The authors declare no conflicts of interest.

## Supporting information




**Supporting Information** brb371328‐sup‐0001‐SuppMat.docx

## Data Availability

The data supporting the findings of this study are available on the CHARLS website (https://charls.pku.edu.cn/).
