# Supplementary material for: Social Isolation Trajectories Spanning Childhood to Adulthood and Mortality Risk in CKM Syndrome: Evidence From CHARLS
Source: Brain Behav. 2026 Mar 30;16(4):e71328. doi: 10.1002/brb3.71328 (PMC13112033; doi:10.1002/brb3.71328)

**Table S1. Baseline characteristics of excluded and included participants**

| **Variables** | **Total** | **Excluded Participants**  **(n = 2,247)** | **Included** **Participants**  **(n = 5,019)** | **SMD** |
| --- | --- | --- | --- | --- |
|  | **(n = 7,266)** |  |  |  |
| **Age, years** | 61.40 ± 9.20 | 64.39 ± 9.89 | 60.06 ± 8.54 | -0.507 |
| **BMI, kg/m²** | 24.48 ± 12.09 | 24.93 ± 20.92 | 24.28 ± 3.95 | -0.164 |
| **Sex, n (%)** |  |  |  |  |
| Female | 3440 (47.34) | 695 (30.93) | 2745 (54.69) | 0.477 |
| Male | 3826 (52.66) | 1552 (69.07) | 2274 (45.31) | -0.477 |
| **Education, n (%)** |  |  |  |  |
| Below elementary school | 3060 (42.11) | 1731 (77.04) | 1329 (26.48) | -1.146 |
| Elementary school | 1668 (22.96) | 207 (9.21) | 1461 (29.11) | 0.438 |
| Above elementary school | 2538 (34.93) | 309 (13.75) | 2229 (44.41) | 0.617 |
| **Residence, n (%)** |  |  |  |  |
| Urban | 1326 (19.56) | 307 (14.67) | 1019 (21.74) | 0.171 |
| Rural | 5454 (80.44) | 1785 (85.33) | 3669 (78.26) | -0.171 |
| **Smoking, n (%)** |  |  |  |  |
| No | 5211 (71.77) | 1760 (78.43) | 3451 (68.79) | -0.208 |
| Yes | 2050 (28.23) | 484 (21.57) | 1566 (31.21) | 0.208 |
| **Drinking, n (%)** |  |  |  |  |
| No | 4721 (65.01) | 1666 (74.24) | 3055 (60.88) | -0.274 |
| Yes | 2541 (34.99) | 578 (25.76) | 1963 (39.12) | 0.274 |
| **CKM, n (%)** |  |  |  |  |
| Stage 1 | 555 (7.64) | 173 (7.70) | 382 (7.61) | -0.003 |
| Stage 2 | 1476 (20.31) | 510 (22.70) | 966 (19.25) | -0.088 |
| Stage 3 | 3586 (49.35) | 1035 (46.06) | 2551 (50.83) | 0.095 |
| Stage 4 | 1649 (22.69) | 529 (23.54) | 1120 (22.32) | -0.029 |
| **Hypertension, n (%)** |  |  |  |  |
| No | 4687 (65.12) | 1419 (63.83) | 3268 (65.70) | 0.039 |
| Yes | 2510 (34.88) | 804 (36.17) | 1706 (34.30) | -0.039 |
| **Diabetes, n (%)** |  |  |  |  |
| No | 6443 (89.19) | 1980 (88.71) | 4463 (89.40) | 0.023 |
| Yes | 781 (10.81) | 252 (11.29) | 529 (10.60) | -0.023 |
| **Dyslipidemia, n (%)** |  |  |  |  |
| No | 5589 (79.1) | 1768 (81.70) | 3821 (77.95) | -0.091 |
| Yes | 1477 (20.9) | 396 (18.30) | 1081 (22.05) | 0.091 |
| **Depression, n (%)** |  |  |  |  |
| No | 4869 (68.29) | 1304 (61.68) | 3565 (71.07) | 0.207 |
| Yes | 2261 (31.71) | 810 (38.32) | 1451 (28.93) | -0.207 |
| **Childhood economic status, n (%)** |  |  |  |  |
| Good | 612 (9.02) | 125 (7.05) | 487 (9.72) | 0.090 |
| Fair | 3510 (51.74) | 852 (48.05) | 2658 (53.04) | 0.100 |
| Poor | 2662 (39.24) | 796 (44.90) | 1866 (37.24) | -0.158 |
| **Childhood health status, n (%)** |  |  |  |  |
| Good | 2545 (37.59) | 614 (34.79) | 1931 (38.57) | 0.078 |
| Fair | 3405 (50.29) | 911 (51.61) | 2494 (49.82) | -0.036 |
| Poor | 821 (12.13) | 240 (13.60) | 581 (11.61) | -0.062 |

Data are presented as mean ± standard deviation (SD) or number (%), as appropriate

An absolute SMD < 0.1 is considered to indicate a negligible difference and good balance between groups

Abbreviations: BMI, body mass index; CKM, cardiovascular-kidney-metabolic syndrome; SMD, standardized mean difference

**Table S2. Methods for evaluating each stage of CKM syndrome**

| **CKM stages** | **Definition** | **Criterion** | **Threshold for CKM conditions** |
| --- | --- | --- | --- |
| **Stage 0:**  **No CKM risk factors** | Individuals with normal BMI and waist circumference, normoglycemia,  normotension, a normal lipid profile, and no evidence of CKD or subclinical or clinical CVD | All criteria are met | BMI ≥25 kg/m^2^ (or ≥23 kg/m^2^ if Asian ancestry) * |
|  |  |  | Waist circumference <88/102 cm in female/male (or if Asian ancestry <80/90 cm in female/male) |
|  |  |  | Participants who do not meet the criteria for other stages |
| **Stage 1:**  **Excess or dysfunctional adiposity** | Individuals with overweight/obesity,  abdominal obesity, or dysfunctional  adipose tissue, without the presence of other metabolic risk factors or CKD | Any of the three criteria is met | Overweight/obesity |
|  |  |  | Abdominal obesity |
|  |  |  | Prediabetes |
|  |  | All criteria are met | SBP <130 mm Hg and DBP <80 mm Hg without self-reported diagnosis of hypertension or use of antihypertensive medications |
|  |  |  | HDL cholesterol <50/40 mg/dL in female/male and triglycerides <150 mg/dL |
|  |  |  | Low-risk CKD in KDIGO classification according to eGFR and UACR: UACR < 30 mg/g and eGFR ≥ 60 ml/min/1.73m^2^. |
|  |  |  | Predicted 10-year CVD risk < 20% |
|  |  |  | No clinical CVD |
| **Stage 2:**  **Metabolic risk factors and CKD** | Individuals with metabolic risk factors (hypertriglyceridemia, hypertension, MetS, diabetes), or CKD | Any of the five criteria is met | Hypertriglyceridemia |
|  |  |  | Hypertension |
|  |  |  | diabetes |
|  |  |  | MetS |
|  |  |  | Moderate-to-high-risk CKD in KDIGO classification |
|  |  | All criteria are met | No very high-risk CKD in KDIGO classification |
|  |  |  | Predicted 10-year CVD risk < 20% |
|  |  |  | No clinical CVD |
| **Stage 3:**  **Subclinical CVD in CKM** | Subclinical CVD among individuals with excess/dysfunctional adiposity, other metabolic risk factors, or CKD | Any of the two criteria is met | Very high-risk CKD in KDIGO classification |
|  |  |  | Predicted 10-year CVD risk ≥ 20% |
|  |  | Any of the eight criteria is met | Overweight/obesity |
|  |  |  | Abdominal obesity |
|  |  |  | Prediabetes |
|  |  |  | Hypertriglyceridemia |
|  |  |  | Hypertension |
|  |  |  | diabetes |
|  |  |  | MetS |
|  |  |  | Moderate-to-high-risk CKD in KDIGO classification |
|  |  | The criterion is met | No clinical CVD |
| **Stage 4:**  **Clinical CVD in CKM** | Clinical CVD among individuals with excess/dysfunctional adiposity,  other metabolic risk factors, or CKD | The criterion is met | Clinical CVD |
|  |  | Any of the nine criteria is met | Overweight/obesity |
|  |  |  | Abdominal obesity |
|  |  |  | Prediabetes |
|  |  |  | Hypertriglyceridemia |
|  |  |  | Hypertension |
|  |  |  | diabetes |
|  |  |  | MetS |
|  |  |  | Moderate-to-high-risk CKD in KDIGO classification |
|  |  |  | Very high-risk CKD in KDIGO classification |

BMI, body mass index; CKD, chronic kidney disease; CKM, cardiovascular-kidney-metabolic; CVD, cardiovascular disease; DBP, diastolic blood pressure; eGFR, estimated glomerular filtration rate; HDL, high-density lipoprotein; KDIGO, The Kidney Disease: Improving Global Outcomes; SBP, systolic blood pressure; UACR, urinary albumin to creatinine ratio.

**Table S3. Variance inflation factors for covariates**

| **Variables** | **VIF** |
| --- | --- |
| **Social isolation trajectories** | 1.016 |
| **Age** | 1.126 |
| **Sex** | 1.459 |
| **Education** | 1.063 |
| **CKM** | 1.122 |
| **Residence** | 1.067 |
| **Smoking** | 1.181 |
| **Drinking** | 1.127 |
| **Hypertension** | 1.106 |
| **Diabetes** | 1.071 |
| **Depression** | 1.05 |
| **Dyslipidemia** | 1.097 |
| **Childhood economic status** | 1.022 |
| **Childhood health status** | 1.016 |
| **BMI** | 1.077 |

Abbreviations: BMI, body mass index; CKM, cardiovascular-kidney-metabolic syndrome

**Table S4. Test of proportional hazards assumption based on Schoenfeld residuals**

| **Variables** | **Chi-square** | ***P*** |
| --- | --- | --- |
| **Social isolation trajectories** | 5.22 | 0.156 |
| **Age** | 1.29 | 0.255 |
| **Sex** | 2.08 | 0.149 |
| **Education** | 0.38 | 0.825 |
| **CKM** | 0.61 | 0.894 |
| **Residence** | 1.24 | 0.266 |
| **Smoking** | 0.23 | 0.634 |
| **Drinking** | 0.14 | 0.708 |
| **Hypertension** | 0.01 | 0.911 |
| **Diabetes** | 0.83 | 0.362 |
| **Depression** | 0.07 | 0.788 |
| **Dyslipidemia** | 0.16 | 0.687 |
| **Childhood economic status** | 0.51 | 0.776 |
| **Childhood health status** | 5.72 | 0.057 |
| **BMI** | 2.71 | 0.1 |
| **GLOBAL** | 20.91 | 0.526 |

Abbreviations: BMI, body mass index; CKM, cardiovascular-kidney-metabolic syndrome

**Table S5. Association of social isolation at different life stages and trajectories with all-cause mortality after excluding deaths within the first 3 years**

| **Variables** | **Model1** | |  | **Model2** | |  | **Model3** | |
| --- | --- | --- | --- | --- | --- | --- | --- | --- |
|  | **HR (95%CI)** | ***P*** |  | **HR (95%CI)** | ***P*** |  | **HR (95%CI)** | ***P*** |
| **Childhood social isolation** |  |  |  |  |  |  |  |  |
| No | Ref |  |  | Ref |  |  | Ref |  |
| Yes | 1.72 (1.34-2.22) | <0.001 |  | 1.41 (1.10-1.82) | 0.009 |  | 1.43 (1.19-1.87) | 0.006 |
| **Adulthood social isolation** |  |  |  |  |  |  |  |  |
| No | Ref |  |  | Ref |  |  | Ref |  |
| Yes | 1.45 (1.04-2.05) | 0.031 |  | 1.24 (0.88-1.76) | 0.140 |  | 1.19 (0.84-1.69) | 0.310 |
| **Social isolation trajectories** |  |  |  |  |  |  |  |  |
| No isolation | Ref |  |  | Ref |  |  | Ref |  |
| Childhood only | 1.72 (1.31-2.27) | <0.001 |  | 1.41 (1.08-1.88) | 0.018 |  | 1.43 (1.07-1.92) | 0.011 |
| Adulthood only | 1.43 (0.89-2.31) | 0.129 |  | 1.25 (0.77-2.03) | 0.370 |  | 1.17 (0.72-1.90) | 0.517 |
| Persistent isolation | 2.17 (1.35-3.51) | <0.001 |  | 1.71 (1.19-2.63) | 0.043 |  | 1.72 (1.19-2.64) | 0.038 |

Model1: Unadjusted

Model2: Adjust for Age, Sex, Education, Residence

Model3: Adjust for Age, Sex, Education, Residence, Smoking, Drinking, CKM, Depression, Childhood economic status, Childhood health status

**Table S6. Association of social isolation at different life stages and trajectories with all-cause mortality after excluding participants with missing covariates**

| **Variables** | **Model1** | |  | **Model2** | |  | **Model3** | |
| --- | --- | --- | --- | --- | --- | --- | --- | --- |
|  | **HR (95%CI)** | ***P*** |  | **HR (95%CI)** | ***P*** |  | **HR (95%CI)** | ***P*** |
| **Childhood social isolation** |  |  |  |  |  |  |  |  |
| No | Ref |  |  | Ref |  |  | Ref |  |
| Yes | 1.67 (1.30-2.15) | <0.001 |  | 1.33 (1.03-1.71) | 0.031 |  | 1.32 (1.02-1.71) | 0.041 |
| **Adulthood social isolation** |  |  |  |  |  |  |  |  |
| No | Ref |  |  | Ref |  |  | Ref |  |
| Yes | 1.52 (1.09-2.13) | 0.008 |  | 1.31 (0.93-1.83) | 0.112 |  | 1.27 (0.90-1.78) | 0.162 |
| **Social isolation trajectories** |  |  |  |  |  |  |  |  |
| No isolation | Ref |  |  | Ref |  |  | Ref |  |
| Childhood only | 1.59 (1.29-2.09) | <0.001 |  | 1.26 (0.95-1.68) | 0.110 |  | 1.24 (0.93-1.66) | 0.153 |
| Adulthood only | 1.31 (0.81-2.12) | 0.264 |  | 1.15 (0.70-1.87) | 0.588 |  | 1.07 (0.66-1.75) | 0.778 |
| Persistent isolation | 2.42 (1.55-3.79) | <0.001 |  | 1.76 (1.12-2.77) | 0.015 |  | 1.77 (1.12-2.80) | 0.014 |

Model1: Unadjusted

Model2: Adjust for Age, Sex, Education, Residence

Model3: Adjust for Age, Sex, Education, Residence, Smoking, Drinking, CKM, Depression, Childhood economic status, Childhood health status

**Figure S1. Proportion of missing values for baseline covariates**


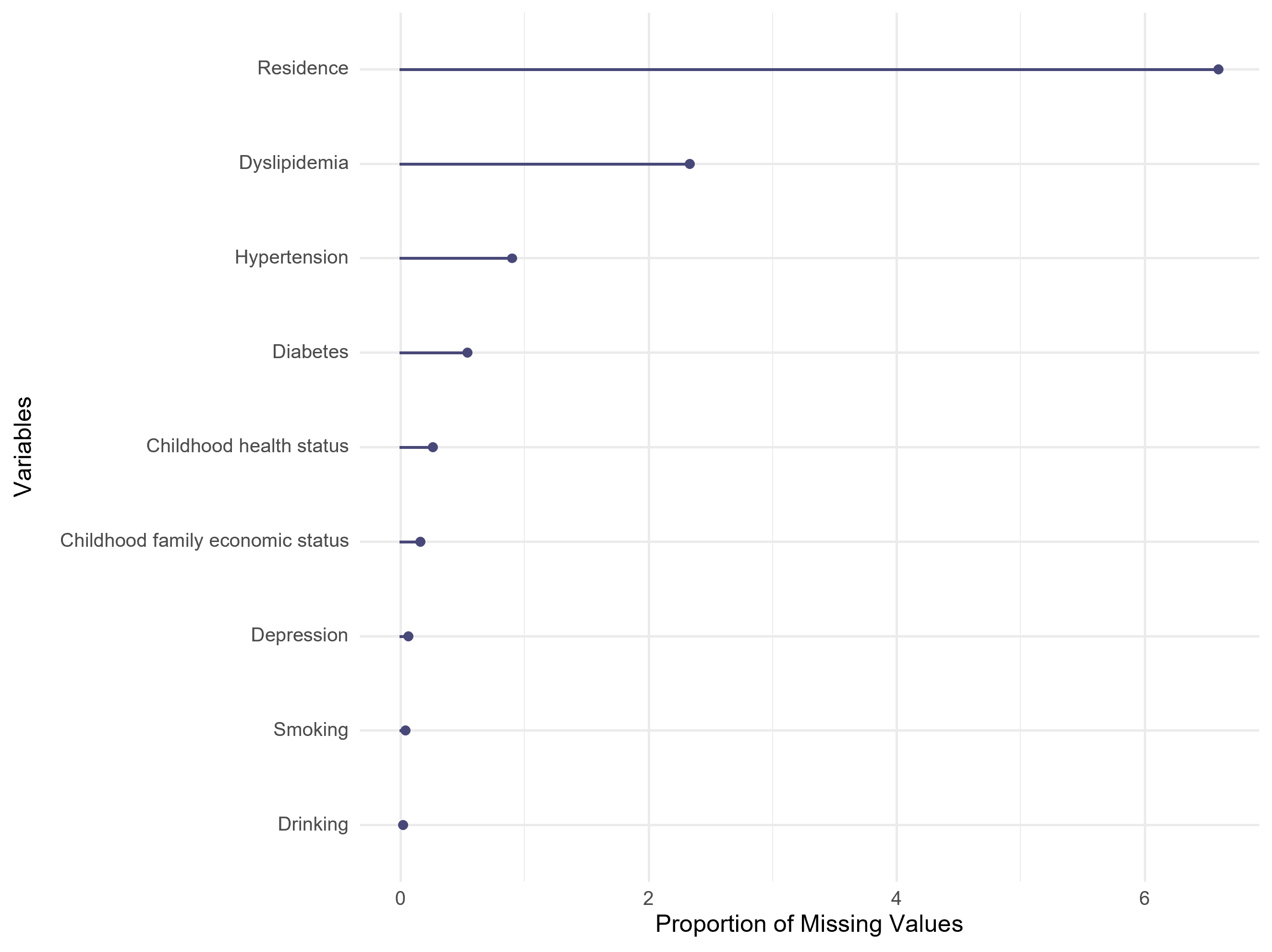


**Figure S2. Proportion of all-cause mortality stratified by social isolation trajectories**


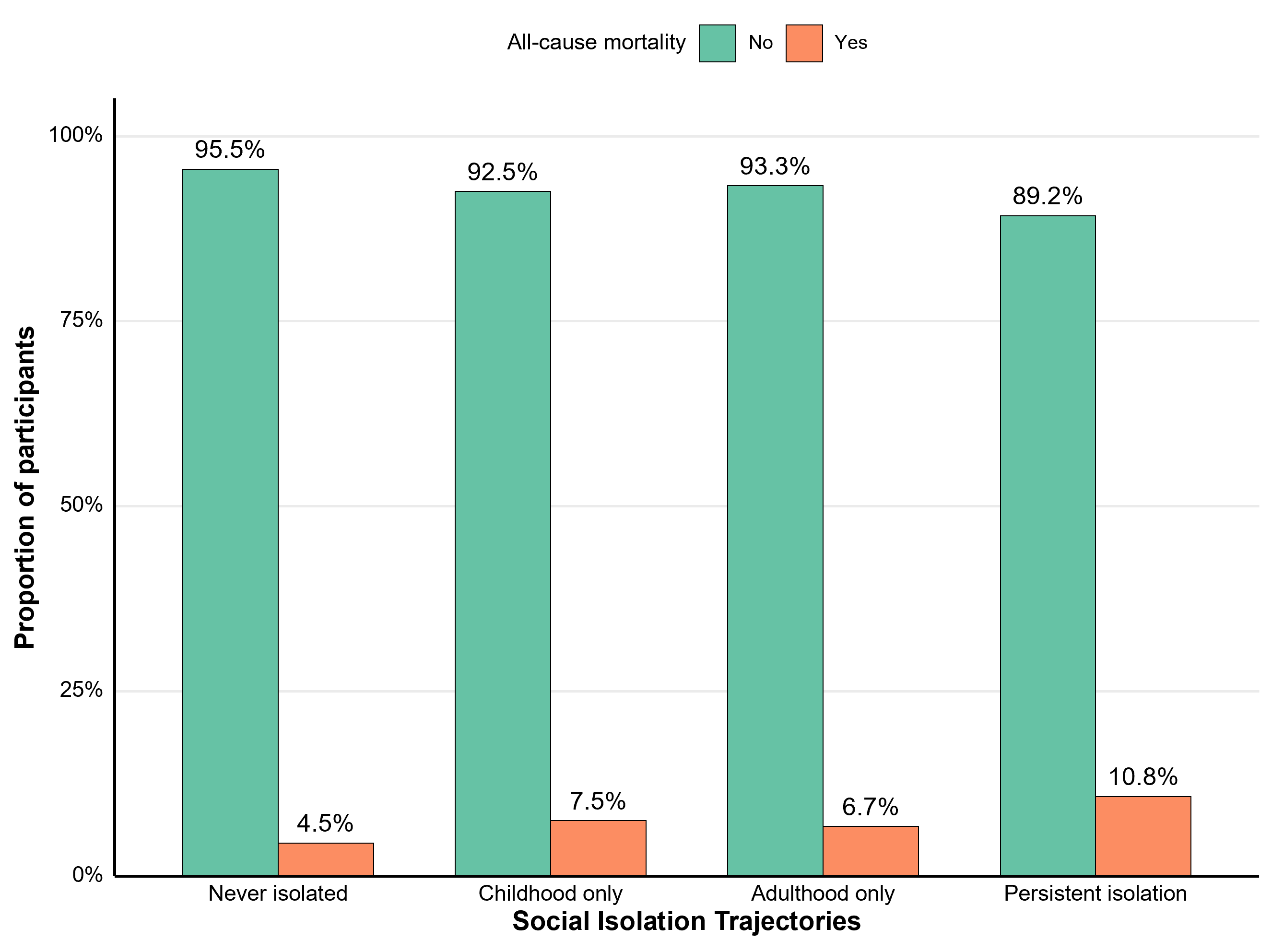


**Figure S3. Kaplan-Meier survival analysis grouped by social isolation trajectories**


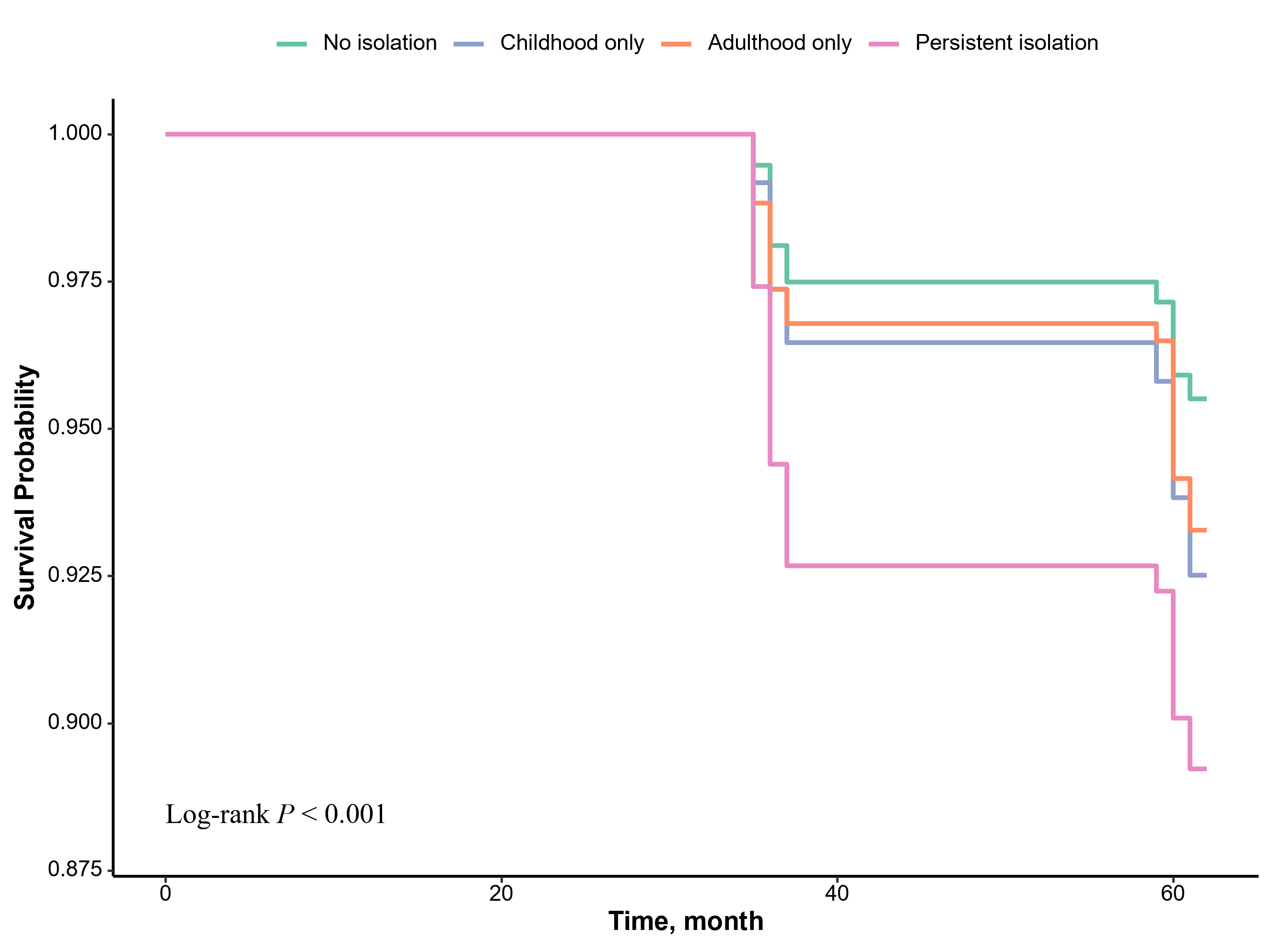

Supplement: Supplementary file 1 — Supporting Information brb371328‐sup‐0001‐SuppMat.docx [file BRB3-16-e71328-s001.docx]
